# Clinical and molecular cytogenetic characterization of a novel 10q interstitial deletion: a case report and review of the literature

**DOI:** 10.1186/s13039-019-0430-8

**Published:** 2019-05-17

**Authors:** John C. Herriges, Sarah L. Dugan, Allen N. Lamb

**Affiliations:** 1 Department of Pathology, University of Utah, Salt Lake City, UT USA; 2 ARUP Laboratories, 500 Chipeta Way, Salt Lake City, UT 84108 USA; 3 Department of Pediatric Medical Genetics, University of Utah, Salt Lake City, USA

**Keywords:** 10q21 deletion, 10q22 deletion, *KAT6B*, *C10ORF11*, SBBYSS, GPS

## Abstract

**Background:**

There are only ten reported cases of interstitial deletions involving cytogenetic bands 10q21.3q22.2 in the literature. Of the ten patients with overlapping 10q21.3q22.2 interstitial deletions, only nine have been characterized by chromosomal microarray analysis. Here, we report a two-and-a-half-year-old patient with a de novo 10.2-Mb deletion that extends from 10q21.3 to 10q22.3 and contains 92 protein coding genes.

**Case presentation:**

The patient is the product of a 37-week dizygotic twin pregnancy and presented with global developmental delay, hypotonia, feeding difficulties, short stature, poor weight gain, scaphocephaly, retrognathia, hypoplasia of the optic nerves/chiasms, a distinctive facial gestalt, as well as additional minor dysmorphic features. The deletion identified in our patient is the second largest reported interstitial deletion involving the 10q21.3q22.2 region. Our patient presents with the generalized features observed in 10q21.3q22.2 deletion patients and also presents with several novel findings including scaphocephaly, hypoplasia of the optic nerves and chiasms, and a very distinctive facial gestalt.

**Conclusions:**

Based on a literature review, we identify a commonly deleted region and suggest that *KAT6B* is a critical gene within the 10q21.3q22.2 region. However, a review of the reported overlapping deletions also suggests that there are additional critical genes contributing to the clinical presentation of these patients.

**Electronic supplementary material:**

The online version of this article (10.1186/s13039-019-0430-8) contains supplementary material, which is available to authorized users.

## Background

Interstitial deletions of the 10q21.3q22.2 region are rarely observed clinically, with only ten patients reported in the literature [1–9]. The initial patient was characterized through G-banded chromosome analysis by Cook et al. (1999), in a 4-month-old male who presented with growth deficiency, developmental delay, ocular hypertelorism, and retrognathia [1]. The nine subsequent cases were characterized by chromosomal microarray (CMA) analysis. These patients have deletions that range in size from 1.36 Mb to 10.4 Mb and include 8 to 88 protein coding genes. Although the size of these deletions is variable, these patients are found to share many of the general features first reported by Cook et al. (1999). However, the specific genes that are associated with these phenotypes have not been thoroughly examined.

In this study, we review the clinical features of the tenth patient with an interstitial deletion involving the 10q21.3q22.2 region that was characterized by CMA. Comparison of our patient to the previously published patients reveals a similar underlying clinical picture, but also identifies several novel clinical features associated with the loss of this region.

## Case presentation

The patient was born following a 37-week dizygotic twin pregnancy. The probands twin was unaffected. At birth, the proband weighed 2250 g (3rd-10th percentile) and was 46 cm long (50th percentile) with an OFC of 34.5 cm (50th–75th percentile). She was hospitalized until 8 days of age for low weight and feeding problems. By 17 months, she was below the 1st percentile for weight, at the 13th percentile for length, and had an OFC in the 65th percentile. At 17 months she was able to bear weight on her feet but was not yet able to pull herself to a stand. She was able to follow simple commands but had no words. At 30 months she was able to pull herself to a stand, was cruising, and had two words as well as the ability to sign a few words. She also had a normal hearing evaluation.

**Fig. 1:**
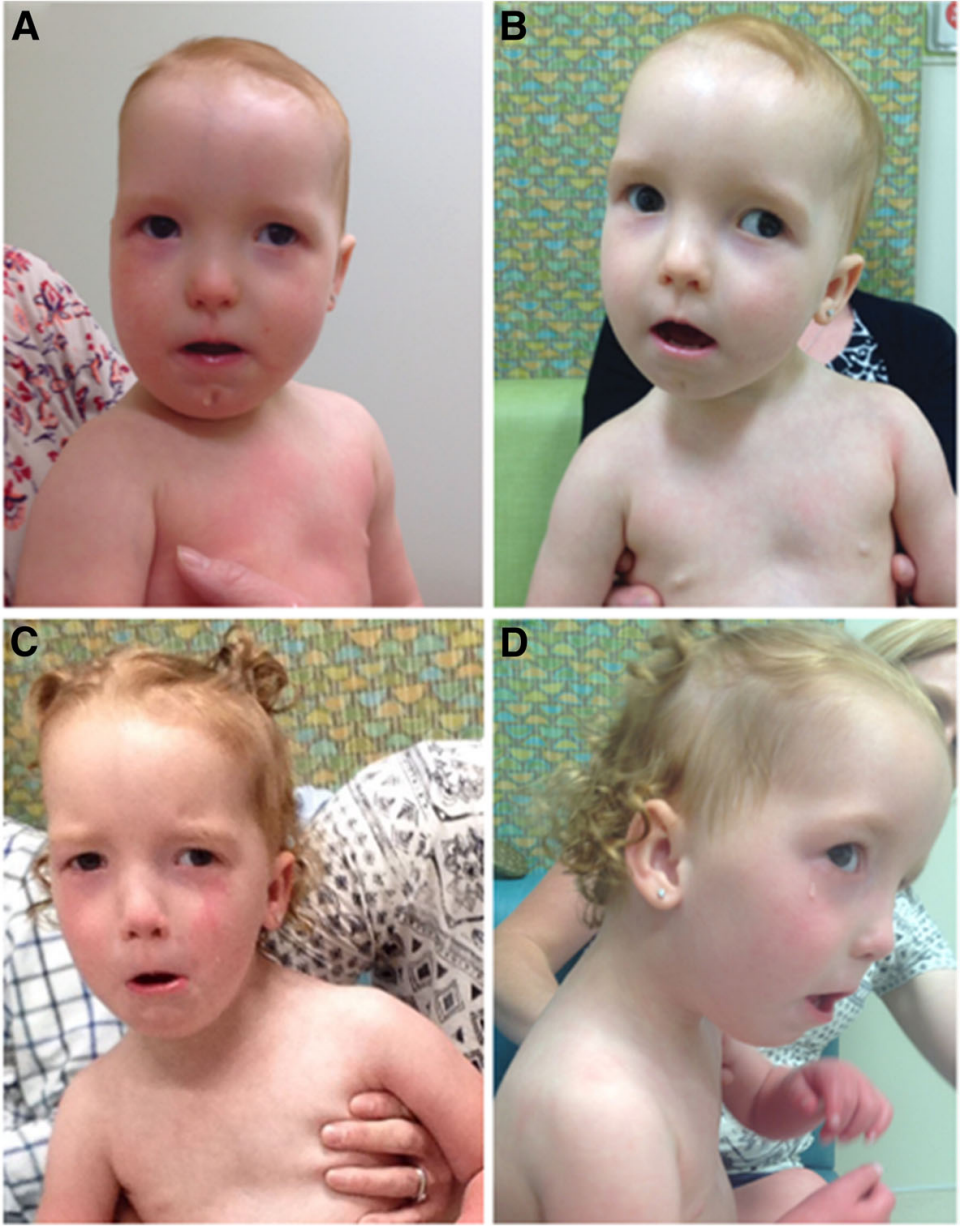
Clinical presentation of the patient. Photographs of the patient at 14 months (**a**), 17 months (**b**), and 30 months (**c**-**d**). The physical features observed in the patient at these visits included: scaphocephaly, telecanthus, retrognathia, and a few additional dysmorphic features

Physical examination at 17 months revealed that the patient was hypotonic (Table 1). She had striking scaphocephaly, a long face, epicanthal folds and telecanthus, and a frontal upsweep (Fig. 1). Depressed nasal ridge with relatively hypoplastic nasal alae and anteverted nares were also noted. Her mouth was narrow, and her upper lip was tented and had a thin vermillion. She had a smooth philtrum, retrognathia, high-arched narrow palate with a bifid uvula, cleft chin, and small posteriorly rotated ears. She was also found to have long and slender fingers and 5th finger clinodactyly. Hands and feet appeared hyperemic. At 30 months she was noted to have flushing of the upper extremities to midway up the forearm and flushing of the legs to the knees. Capillary refill was normal.

**Table 1:** Summary of the clinical findings in the reported patients with overlapping 10q21.3q22 deletions

	Tzschach et al. 2011 Patient 1 [8]	Tzschach et al. 2011 Patient 2 [8]	Tzschach et al. 2011 Patient 3 [8]	Reddy et al. 2011 Case 1 [5]	Lei et al. 2016 [3]	Gannon et al. 2015 (DECIPHER: 258813) [2]	Shimojima et al. 2017 [6]	Preiksaitiene et al. 2017 [4]	Current Patient	Bartnik et al. 2014 Patient 50 [9]
Karyotype	46,XX,del(10)(q21.3q22.2)	46,XX,del(10)(q22.2q22.3)	46,XX,del(10)(q22.2q22.3)	46,XX,del(10)(q22.1q22.2)	46,XX	46,XY	46,XX	NA	NA	NA
Deleted region [GRCh37]	chr10:69659286–77,597,174	chr10:75527732–78,793,670	chr10:75379383–79,041,376	chr10:74445789–77,407,019	chr10:76652946–78,419,911	chr10:75971593–78,526,861	chr10:64892035–75,320,005	chr10:74236933–79,422,266	chr10:68735254–78,885,714	chr10:69295658–70,664,371
Size of deletion	7.9 Mb	3.2 Mb	3.6 Mb	2.96 Mb	1.77 Mb	2.5 Mb	10.4 Mb	5.2 Mb	10.2 Mb	1.36 Mb
Protein coding genes	88	19	23	35	8	9	73	37	92	15
Age at examination	3 Years 9 months	4 Years 6 months	2 Years 6 months	3 Years	2 Years 6 months	11 Years	3 Years	4 Years	2 Years 6 months	14 Years
Sex	Female	Male	Female	Female	Female	Male	Female	Female	Female	Male
Delayed Psychomotor development	+	+	+	+	+	UK	+	+	+	+
Speech Delay	+	+	+	+	+	+	+	+	+	UK
Growth Deficiency	+	–	+	+	+	UK	+	–	+	+
Feeding difficulties	+	–	–	–	+	UK	+	+	+	+
Muscular Hypotonia	+	–	+	–		+	+	+	+	+
OFC	25-50th Centile	75-90th Centile	10th Centile	75-90th Centile	10-50th Centile	Macrocephaly	−2.0 SD	75th Centile	65th Centile	10–25 Centile (at birth)
Ocular	Hypertelorism	Hypertelorism	Hypertelorism, strabismus, telecanthus, epicanthic inversus, ptosis	Telecanthus	–	Telecanthus, blepharophimosis, short palpebral fissures and prominent epicanthic folds	Hypertelorism, downslanting palpebral fissures, and epicanthal folds	Bilateral bepharophimosis, telecanthus, epicanthic inversus, ptosis,	Telecanthus	Strabismus, nystagmus
Ears	Low set, posteriorly rotated	Low set	–	Small, slightly low set, posteriorly rotated depressor	–	Small ears	Low set	Small ears	Small, posteriorly rotated	UK
Mouth	Dental lamina cysts	Small mouth		Small mouth, oral asymmetry, high-arched palate	Cleft Palate	Normal Palate	UK	Small mouth, long philtrum,	Tented upper lip, thin vermillion, smooth philtrum, high-arched and narrow palate, bifid uvula	High palate, short philtrum,
Retrognathia	+	+	–	‘Small chin’	–	UK	UK	–	+	UK
Digital findings	Long and lean thumbs	Fifth finger clinodactyly	–	–		UK	UK	Fifth finger clinodactyly	Fifth finger clinodactyly, Long slender fingers	Brachydactyly
Genital Anomalies	–	–	Small labia minora	Hypoplastic external genitalia		UK	UK	–	–	UK
MRI findings	Delayed myelination	–		–	–	Mild frontal atrophy	UK	Mild internal hydrocephalus; arachnoidal cyst	Small optic nerves, delayed myelination of the optic radiations	Focal perinatal ischaemic/an-oxemic hypomyelination changes
Other	Unilateral deafness, gastroesophageal reflux, long and lean thumbs	Brachycephaly	Hypoplasia of the midface	IUGR, Facial Palsy	Mild hearing impairment, feeding difficulties	IUGR	Tetrology of Fallot, atresia of the pulmonary artery, PDA, and atrial septal defect; flat nasal bridge; small hands and feet, webbed neck, low hairline,	“Mask like face”, ventricular septal defect, hoarse voice	Scaphocephaly depressed nasal ridge	Congenital heart defect (VSD and PDA), Hirschsprung disease, wide nasal bridge, large nose with bulbous tip, malocclusion

UK: Unknown; −: Absent; +: Present; IUGR: Intrauterine growth restriction; SD: standard deviations; PDA: Patent ductus arteriosus; VSD: Ventricular Septal Defect; MRI: Magnetic resonance imaging; OFC: Occipitofrontal Circumference

Additional studies included a normal echocardiogram and renal ultrasound, as well as a head CT that showed no evidence of craniosynostosis. On dilated eye exam she was noted to have small optic nerves. Strabismus and lacrimal duct stenosis were also noted, and she had surgical treatment for both these issues. An MRI of the brain later confirmed the small optic nerves, and also showed a small chiasm and delayed myelination of the optic radiations.

## Methods and results

Copy number and SNP-genomic microarray analysis was preformed using the Affymetrix CytoScan HD microarray platform. This analysis showed a 10.2 Mb deletion on chromosome 10 that involved 10q21.3 to 10q22.3 (arr[CRCh37] 10q21.3q22.3: 68735254_78,885,714) and included 92 protein coding genes (Additional file 1: Figure S1A). No other significant DNA copy number changes or copy-neutral long continuous stretches of homozygosity were detected.

Parental metaphase FISH analysis was performed with the RP11-227H15 clone, which localizes to 10q22.1 (Empire Genomics) and a chromosome 10 centromeric probe (Cep10; Abbott, Inc) (Additional file 1: Figure S1B-E). The proband’s sample showed loss of the RP11-227H15 signal at 10q22 (Additional file 1: Figure S1C). In the parental samples, both probes co-localized to chromosome 10 (Additional file 1: Figure S1D, E). These results are consistent with the proband’s deletion representing a de novo event.

Written informed consent was obtained from the patient’s parents to be involved in this case report and for the publication of photographs.

## Discussion and conclusions

**Fig. 2:**
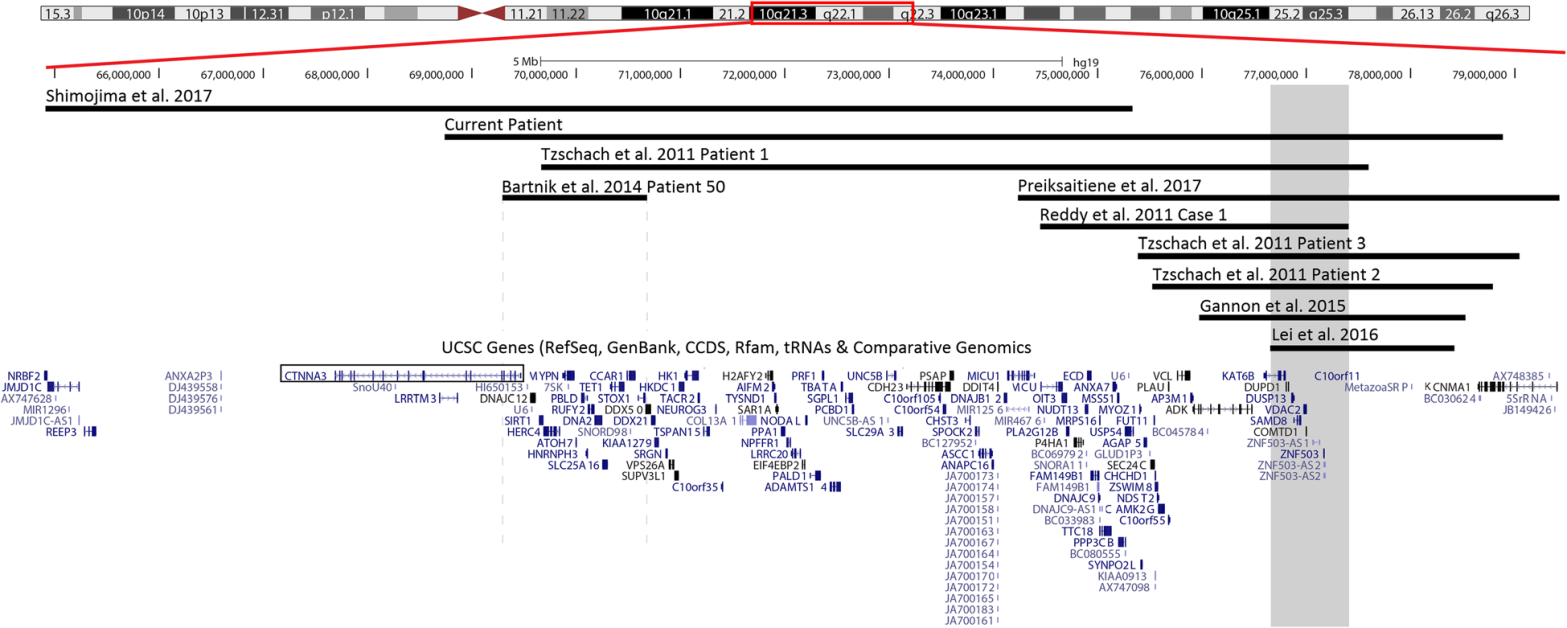
Overlap map of 10q21.3q22 deletions. The deletion identified in our patient is the second largest reported deletion in this region that has been characterized by chromosomal microarray. Comparison of the reported deletions reveals a 754 kb overlapping deleted region (gray) that is shared by eight of the ten patients. Dashed gray lines represent the breakpoints of patient 50 in Bartnik et al. (2014) [9]. The *CTNNA3* open reading frame is boxed in. Note: Patients characterized by G-banded chromosome analysis are not depicted in this figure due to the uncertainty of the deleted region

There are currently nine patients reported in the literature with interstitial deletions in the 10q21.3q22.2 region characterized by CMA, [2–9]. These deletions range in size from 1.36 Mb to 10.4 Mb and include 8 to 88 protein coding genes (Table 1). Our patient carries the second largest reported interstitial deletion of this region and shares many non-specific clinical features with the previously reported patients with deletions characterized by CMA (Fig. 2 and Table 1). These features include: developmental delay (speech and/or psychomotor) (8/8), growth deficiency (6/8), feeding difficulties (5/8), and hypotonia (6/8) (Table 1). She also has several minor anomalies of the face that overlap those described in the reported patients, including micro/retrognathia (3/9), small mouth (3/9), posteriorly rotated ears (2/9), and telecanthus (4/9) (Table 1). However, the scaphocephaly, hypoplasia of the optic nerves and chiasms, and distinctive facial gestalt, which were observed in our patient, have not been described in any of the other published deletion patients.

There is no region of overlap that is shared among all of the reported deletions; however, eight of the ten CMA cases, including the current patient, share a 754 kb region of overlap within 10q22.2 (Fig. 2). The common region includes eight protein-coding genes (*KAT6B*, *DUPD1*, *DUSP13*, *SAMD8*, *VDAC2*, *COMTD1*, *ZNF503*, and *C10ORF11*), six of which are OMIM genes (Additional file 2: Table S1). Each of the eight protein-coding genes has been shown to be evolutionarily conserved in mice, suggesting they may potentially be significant for development. To the best of our knowledge, the functional significance of these genes during mouse development has only been examined in vivo for *Vdac2*, *Samd8*, and *Kat6b*. Unfortunately, the phenotypic information associated with loss of *Vdac2* and *Samd8* is limited, with *Vdac2* only being associated with promoting thymocyte survival and loss of *Samd8* not being associated with any overt phenotype [10, 11]. However, loss of *Kat6b* has been associated with failure to thrive, craniofacial abnormalities, feeding difficulties, abnormal closure of the skull sutures, and abnormal brain development in mice [12, 13]. In humans, only two these eight protein coding genes have been previously associated with a clinical phenotype, *C10ORF11* and *KAT6B* .

*C10ORF11* (also known as *LRMDA*) is a little-studied gene that encodes a protein involved with melanocyte differentiation [14]. Previous studies have proposed that loss of *C10ORF11* is associated with the developmental delays observed in patients with 10q21.3q22.2 deletions [8]. This proposed association was based on the smallest region of overlap (SRO) of the three initial reported patients, and a single patient with a de novo reciprocal translocation that interrupts *C10ORF11* after exon 4 (of 6 total exons) [8]. However, a subsequent study has found that truncating mutations in *C10ORF11* (frameshift and nonsense) are associated with autosomal recessive albinism, with no reported developmental delays [14]. Therefore, the data supporting the association of *C10ORF11* with developmental delays in 10q21.3q22.2 deletion patients is equivocal and will require more data to confirm.

*KAT6B* (previously known as *MORF*, *QKF*, and *MYST4*) encodes a transcriptional coactivator protein with histone acetyltransferase activity [15]. In humans, *KAT6B* is expressed throughout the developing fetus, and its strongest expression is within the fetal brain [16]. The initial clinical report on a *KAT6B* mutation was a reciprocal translocation which interrupted *KAT6B* after the third intron [16]. The patient who carried this rearrangement was described as having a “Noonan syndrome-like phenotype” with mild intellectual disability, short stature, microcephaly, and a distinct facial gestalt (including blepharophimosis, ptosis, retrognathia, and a high-arched palate). Since that study, multiple groups have identified that sequence level alterations (nonsense, missense, and frameshift) in *KAT6B* are associated with a spectrum of autosomal dominant conditions including Genitopatellar syndrome (GPS) (OMIM: 606170) and a variant of Ohdo syndrome known as Say-Barber-Biesecker-Young-Simpson syndrome (SBBYSS) (OMIM: 603736) (Additional file 2: Table S1) [2, 17–20]. The major clinical characteristics of GPS include skeletal anomalies, flexion contractures, microcephaly, corpus callosum agenesis, absent or hypoplastic patellae, and renal anomalies [21]. SBBYSS is associated with a distinctive facial gestalt (“expressionless or mask-like face”, small mouth, thin upper lip, and ptosis), long thumbs, lacrimal duct abnormalities, and patellar hypoplasia or agenesis. In addition, both SBBYSS and GPS are associated with developmental delay/intellectual disability, hypotonia, congenital heart defects, feeding difficulties, dental anomalies, hearing loss, and genital abnormalities (Additional file 2: Table S1) [18, 20].

The phenotypic similarity of patients who carry deletions involving the 754 kb region of overlap to GPS/SBBYSS patients suggests that *KAT6B* may be a critical gene within this region. The common features associated with deletions involving the region of overlap and this gene include developmental delay (speech and psychomotor), hypotonia, feeding difficulties, poor weight gain, micro/retrognathia, and additional minor dysmorphic features (Table 1). All of these findings have been reported in patients with both SBBYSS and GPS, and with the exception of micro/retrognathia, are more commonly observed in SBBYSS patients [20]. The basis for the stronger overlap in clinical presentation of SBBYSS patients with 10q21.3q22.2 deletions involving *KAT6B* is unknown. However, it has been hypothesized that *KAT6B* haploinsufficiency is associated with SBBYSS, whereas *KAT6B* dominant-negative/gain-of-function-type alleles are associated with GPS [2, 17]. This would explain why none of the 10q21.3q22.2 deletion patients have any of the major clinical findings that are uniquely associated with GPS (Table 1), and why their features are more consistent with SBBYSS [2, 17]. However, this does not clarify why only one of the patients with a *KAT6B* whole gene deletion is reported to have the typical “SBBYSS facial gestalt” [4]. One hypothesis for this observation is that even the smallest reported 10q21.3q22.2 deletion involving *KAT6B* also involves seven other genes (Fig. 2). It is possible that the loss of these additional genes may alter the clinical features observed in SBBYSS patients. Alternatively, this may suggest that the disease mechanism associated with SBBYSS is more complicated than haploinsufficiency. Additional studies are still needed to explore these two hypotheses, including functional studies that look at the precise disease mechanisms associated with the GPS-SBBYSS disease spectrum, as well as more focal deletions involving *KAT6B*.

The two deletions in 10q21.3q22.2 that do not involve *KAT6B* are the patient in Shimojima et al., (2017), who has the largest interstitial deletion in this region, and patient 50 in Bartnik et al., (2014), whose deletion localizes primarily to 10q21.3 (Fig. 2; Table 1) [6, 9]. These two deletions overlap the proximal portion of the deletion identified in our patient and suggest that *KAT6B* is not the only significant gene within our patient’s deletion. However, due to the significant size discrepancy of these two deletions (Fig. 2), and their general/non-specific clinical features (Table 1), it is difficult to determine what the other critical genes are.

There are several genes within these two deletions that have been associated with abnormal clinical findings, but none that are clearly causative. *CTNNA3* is of particular interest with regards to the neuronal findings observed in 10q21.3q22.2 deletion patients, as it is expressed within the brain, and has been associated with autism spectrum disorder [22]. However, the clinical significance of *CTNNA3* deletions is unclear, as *CTNNA3* exon level deletions are also frequently observed in control populations [22]. Heterozygous sequence level variants in *DNA2* have been associated with autosomal dominant pediatric and adult onset hypotonia, ptosis, and limb-girdle muscle weakness [23]. Hypotonia was reported in both of the patients with deletions not involving *KAT6B*, suggesting that the loss of *DNA2* may be associated with this finding (Fig. 2: Table 1). However, it is unknown whether whole gene deletions of *DNA2* would have the same functional consequence as the reported sequence level changes; therefore, the significance of this association is uncertain. Finally, sequence level variants in *MYPN* have been associated with cardiomyopathy [24]. This finding has not been reported in any of the patients with a deletion of this gene, and like *DNA2*, it is unknown whether a whole gene deletion would have the same functional consequence as the reported variants. Nonetheless, these are potential candidate genes that may contribute in part to the findings observed in patients with deletions in this region.

The unique clinical findings observed in our patient include scaphocephaly and hypoplasia of the optic nerves and chiasms. A survey of the genes within the proband’s 10.2 Mb deletion (10q21.3q22.3) did not identify any genes that are known to be causative of these phenotypes. However, in mice, *Kat6b* has been shown to regulate skull suture closure [16]. In addition, recent studies in animal models have suggested that *ZNF503* (also known as *NOLZ1*), regulates neuronal development through the retinoic acid pathway [25, 26]. This pathway is a known mediator of optic nerve development, which suggests that *ZNF503* may be associated with the hypoplastic optic nerve that was observed in our patient [27]. Both of these genes lie within the 754 kb region of overlap found in seven of the nine other 10q21.3q22.2 deletion patients characterized by CMA (Fig. 2). Therefore, if these two genes are associated with these findings, it is likely a low penetrance phenotype.

Of note, recent studies have also suggested that chromatin structure, or topological associated domains (TADs), is an important mediator of gene expression [28, 29]. Since deletions and duplications can alter the relationship between genes and their regulatory elements, the disruption of TADs could explain why different size deletions in the same region of the genome may result in patients showing apparent variability in clinical presentation, without having significant differences in the affected gene content. For this 10q21.3q22.2 region, it appears likely that *KAT6B* is the critical gene. However, additional functional studies are still needed to examine whether the deletion observed in our patient affects any TADs that may alter the expression of genes outside of the deleted region, as this could account for some of the unique features that she presented with.

In summary, we present the tenth patient with a deletion in this region that was characterized using CMA. The reported patients with deletions of this region are found to have a similar clinical presentation that includes developmental delay, hypotonia, growth deficiency, ocular anomalies, retrognathia, and craniofacial defects. Comparison of the overlapping deletions found in the reported patients identified a 754 kb region of overlap in eight of the ten patients, which contains eight protein-coding genes, including *KAT6B*. Given the overlapping clinical presentation of SBBYSS/GPS and 10q21.3q22.2 deletion patients, we suggest that *KAT6B* is a critical haploinsufficient gene within 10q21.3q22.2. However, the large number of genes in most of these cases makes correlations difficult and additional patients with smaller deletions are needed to further clarify the significance of individual genes in the 10q21.3q22.2 region.

## Additional files

Additional file 1: **Figure S1. A-E**: Testing results (**A**) The chromosomal microarray analysis showed a 10.2 Mb interstitial deletion involving chromosome 10 from 10q21.3 to 10q22.3. (**B**) FISH testing was performed using the CEP10 (centromere) and RP11-227H15 (10q22) probes. The proband (**C**) showed a loss of the RP11-227H15 probe at 10q22, while the maternal (**D**) and paternal (**E**) samples showed co-localization of both probes to chromosome 10. (TIF 1710 kb)

Additional file 2: **Table S1.** OMIM genes in the overlapping deleted region on 10q22.2. List of the OMIM protein coding genes found with the minimal deleted region and their associated clinical presentations. (DOCX 14 kb)

## Abbreviations

CMA: Chromosomal microarray
GPS: Genitopatellar syndrome
IUGR: Intrauterine growth restriction
MRI: Magnetic resonance imaging
OFC: Occipitofrontal Circumference
PDA: Patent ductus arteriosus
SBBYSS: Say-Barber-Biesecker-Young-Simpson syndrome
SD: Standard deviations
TAD: Topological Associated Domain
VSD: Ventricular Septal Defect
