# Supplementary figures and images for: Clinical and molecular cytogenetic characterization of a novel 10q interstitial deletion: a case report and review of the literature
Source: Mol Cytogenet. 2019 May 17;12:20. doi: 10.1186/s13039-019-0430-8 (PMC6525357; doi:10.1186/s13039-019-0430-8)

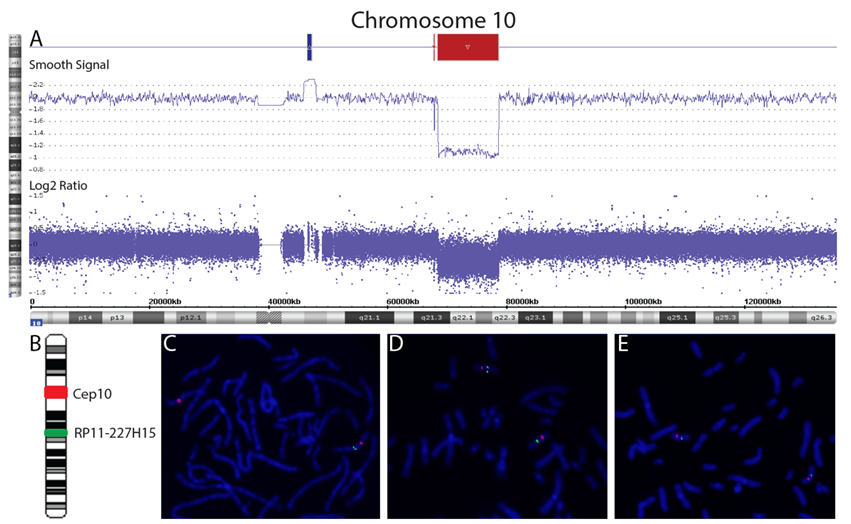

Supplement: Supplementary file 1 — Figure S1. A-E: Testing results (A) The chromosomal microarray analysis showed a 10.2 Mb interstitial deletion involving chromosome 10 from 10q21.3 to 10q22.3. (B) FISH testing was performed using the CEP10 (centromere) and RP11-227H15 (10q22) probes. The proband (C) showed a loss of the RP11-227H15 probe at 10q22, while the maternal (D) and paternal (E) samples showed co-localization of both probes to chromosome 10. (TIF 1710 kb) [file 13039_2019_430_MOESM1_ESM.tif]
