# Supplementary material for: Clinical and molecular cytogenetic characterization of a novel 10q interstitial deletion: a case report and review of the literature
Source: Mol Cytogenet. 2019 May 17;12:20. doi: 10.1186/s13039-019-0430-8 (PMC6525357; doi:10.1186/s13039-019-0430-8)
Supplement: Supplementary file 2 — Table S1. OMIM genes in the overlapping deleted region on 10q22.2. List of the OMIM protein coding genes found with the minimal deleted region and their associated clinical presentations. (DOCX 14 kb) [file 13039_2019_430_MOESM2_ESM.docx]

Additional file 2: Table S1: OMIM genes in the overlapping deleted region on 10q22.2

| OMIM ID | Gene | | Associated Disorder | Inheritance | Clinical Presentation |
| --- | --- | --- | --- | --- | --- |
|  | Symbol | Title |  |  |  |
| 605880 | KAT6B | Lysin Acetyltransferase 6B (Histone acetyltransferase) | SBBYSS syndrome | Autosomal Dominant | Major Features: Long thumbs/great toes, immobile mask-like face (expressionless face), blepharophimosis/ptosis, lacrimal duct anomalies, and patellar hypoplasia/agenesis Minor Features: Congenital heart defect, dental anomalies, hearing loss, thyroid anomalies, anal anomalies, hypotonia, and global developmental delay/intellectual disability |
|  |  |  | Genitopatellar syndrome | Autosomal Dominant | Major Features: Genital anomalies, Patellar hypoplasia/agenesis, flexion contractures at the hips and knees, club feet, agenesis of the corpus callosum, microcephaly, hydronephrosis and/or multiple renal cysts Minor Features: Congenital heart defect, Dental anomalies, hearing loss, thyroid anomalies, anal anomalies, hypotonia, and global developmental delay/intellectual disability |
| 613191 | DUSP13 | Dual-specificity phosphatases 13 | None | NA | NA |
| 611575 | SAMD8 | Sterile Alpha Motif Domain-containing Protein 8 | None | NA | NA |
| 193245 | VDAC2 | Voltage-Dependent Anion Channel 2 | None | NA | NA |
| 613902 | ZNF503 | Zinc Finger Protein 503 | None | NA | NA |
| 614537 | C10ORF11 | Chromosome 10 Open Reading Frame 11 | Albinism, oculocutaneous | Autosomal recessive | Abnormal pigmentation, photophobia, and nystagmus |

NA- Not applicable
